# An explanatory randomised placebo controlled trial of levothyroxine supplementation for babies born <28 weeks’ gestation: results of the TIPIT trial

**DOI:** 10.1186/1745-6215-14-211

**Published:** 2013-07-11

**Authors:** Sze M Ng, Mark A Turner, Carrol Gamble, Mohammed Didi, Suresh Victor, Donal Manning, Paul Settle, Richa Gupta, Paul Newland, Alan Michael Weindling

**Affiliations:** 1 Department of Women’s and Children’s Health, Institute of Translational Medicine, University of Liverpool, Liverpool, UK; 2 Clinical Trials Research Centre, University of Liverpool, Liverpool, UK; 3 Department of Endocrinology, Alder Hey Children’s Foundation Trust, Liverpool, UK; 4 Developmental Biomedicine Research Group, Manchester Academic Health Sciences Centre, University of Manchester, Manchester, UK; 5 Neonatal Unit, Wirral Teaching Hospital Foundation Trust, Wirral, UK; 6 Neonatal Unit, Salford Royal NHS Foundation Trust, Salford, UK; 7 Neonatal Unit, Royal Preston Hospital, Preston, UK; 8 Department of Biochemistry, Alder Hey Children’s Foundation Trust, Liverpool, UK; 9 Department of Paediatrics, Southport and Ormskirk NHS Trust, Wigan Road, Ormskirk, Lancashire L39 2AZ, UK

**Keywords:** Thyroxine supplementation, RCT, Extreme preterm, Brain, Growth

## Abstract

**Background:**

Babies born before 28 weeks’ gestation have lower plasma thyroid hormone concentrations than more mature infants. This may contribute to their risk of poor developmental outcome. Previous studies have suggested that thyroxine supplementation for extremely preterm neonates may be beneficial. The aim of this study was to investigate the effect of administration of supplemental thyroxine to very premature babies on brain size and somatic growth at 36 weeks’ corrected gestational age (CGA).

**Methods:**

In this explanatory multicentre double blind randomised placebo controlled trial, 153 infants born below 28 weeks’ gestation were randomised to levothyroxine (LT4) supplementation or placebo until 32 weeks’ CGA. The primary outcome was brain size assessed by the width of the subarachnoid space measured by cranial ultrasound at 36 weeks’ CGA. Lower leg length was measured by knemometry.

**Results:**

Babies in the LT4-supplemented and placebo groups had similar baseline characteristics. There were no significant differences between infants given LT4 (*n*=78) or placebo (*n*=75) for width of the subarachnoid space, head circumference at 36 weeks’ CGA, body weight at 36 weeks’ CGA or mortality. Infants who received LT4 had significantly shorter leg lengths at 36 weeks’ CGA although adjusted analysis for baseline length did not find a statistical difference. There was a significant correlation between low FT4 and wider subarachnoid space. No unexpected serious adverse events were noted and incidence of adverse events did not differ between the two groups.

**Conclusion:**

This is the only randomised controlled trial of thyroxine supplementation targeting extremely premature infants. Supplementing all babies below 28 weeks’ gestation with LT4 had no apparent effect on brain size. These results do not support routine supplementation with LT4 for all babies born below 28 weeks’ gestation.

**Trial registration:**

Current Controlled Trials ISRCTN89493983

EUDRACT number: 2005-003-09939

## Background

Hypothyroidism causes neurodevelopmental disability in untreated congenital hypothyroidism babies [1,2] and this is preventable by supplementary levothyroxine (LT4) [3]. The proportion of preterm infants with low plasma free thyroxine (FT4) concentrations increases with decreasing gestational age [4,5]. Up to 69% of infants born below 30 weeks’ gestation have lower thyroxine (FT4) concentrations than term babies [5-9]. However, LT4 supplementation does not invariably improve neurodevelopmental outcome [6,10-14]. It is unclear whether the condition known as transient hypothyroxinaemia of prematurity (THOP) [15], defined by low FT4 concentrations and normal concentrations of thyroid stimulating hormone (TSH), contributes to the causation of neurodisability. A Cochrane review [16] of thyroid hormone supplementation for premature infants concluded that future trials of sufficient size are needed to detect clinically important differences in neurodevelopmental outcome. The studies should enrol those infants most likely to benefit from thyroid hormone therapy such as extremely preterm infants. There is therefore clinical uncertainty about whether apparently low plasma FT4 concentrations should be treated or whether they are a marker of illness severity or a normal concomitant of prematurity.

The largest study to date has been a randomised controlled trial of levothyroxine (LT4) supplementation by van Wassenaer *et al.*[8,14] that recruited 200 infants on the basis of gestational age (<30 weeks’ gestation). There were no differences in outcomes for the whole sample. However, a *post hoc* analysis of only 46 infants <27 weeks’ gestation (19 treated with LT4, 27 with placebo) showed an improved Bayley Scales II Mental Development Index (MDI; effect size 0.74 of SD) at 2 years. An improved IQ score (effect size 0.65 of SD) at age 5 years [17] was observed among the most immature infants treated with thyroxine. The reliability of that observation was limited by the small number of infants in the subgroup analysis, which had not been pre-specified. In other studies with between 31 and 49 participants, thyroid hormone supplementation for preterm infants under 32 weeks’ gestation had either beneficial or no apparent effects [6,8,13,18,19]. If the effect seen in the *post hoc* analysis by van Wassenaer *et al.*[14] was confirmed in larger studies, LT4 supplementation would give a clinically relevant improvement to clinical outcome. Accordingly, it is essential to examine whether this effect could be reproduced in a larger sample. We chose to power the study by a surrogate outcome in order to determine whether future studies powered on neurodevelopmental outcomes are warranted.

This study was designed as an explanatory trial [20] aimed at determining whether supplementation with LT4 postnatally until 32 weeks’ corrected gestational age (CGA) affects brain growth, somatic growth, the hypothalamic-pituitary-thyroid axis and hypothalamo-pituitary-adrenal axis. T4-supplementation was continued until 32 weeks’ CGA because there is epidemiological evidence from neurodevelopmental data acquired in the UK and Bavaria, Germany, that development from 32 weeks post conception until term-equivalence is fairly constant, whereas at lower gestations there is a loss of around 5 IQ points for each week of gestation [21]. Thus, infants below 32 weeks gestation appear to be particularly vulnerable to neurodevelopmental impairment and therefore most likely to benefit from any intervention. Transient hypothyroxinaemia was chosen because there are data to suggest that this intervention may be useful and the choice of LT4 supplementation and its dose was based on the previous study of van Wassenaer *et al*. [14].

## Methods

This was a randomized, double blind, placebo-controlled, explanatory trial of postnatal LT4 supplementation given until 32 weeks’ CGA (that is, gestation plus postnatal age) to infants born below 28 weeks’ gestation. The protocol has been published [22].

### Subjects

Multiple births were included with both babies randomized to the same arm. The following were excluded: infants born to mothers with known thyroid disease, on antithyroid medications or amiodarone; infants with major congenital or chromosomal abnormalities known to affect thyroid function or brain development; infants whose mothers died within 5 days of giving birth.

### Intervention

Two forms of the active medication with corresponding placebos were used: intravenous LT4 (Levothyroid, Aventis Pharma, Spain, Marketing Authorization 971622) and oral LT4 solution (Evotrox, Kappin Ltd, London, Marketing Authorization PL20249/0007). The LT4-supplemention regime used the same dosage regimen as previous studies [14,23], that is 8 μg/kg birthweight/day as a single daily dose. Either intravenous LT4 or placebo (5% dextrose) was started during the first 5 days after birth. Once enteral feeds were fully established, the oral solution Evotrox or placebo (carrier solution without active drug) were given daily until 32 weeks’ CGA.

### Randomisation

Randomisation codes were computer generated using STATA with random variable block sizes of two and four, stratified by centre and gestation at birth (< 25 weeks’ gestation, 25-26^+6^ weeks’ gestation, 27-27^+6^ weeks’ gestation). Allocation concealment was done by the pharmacy departments of participating hospitals. Parents, care providers and outcome assessors were unaware of treatment allocation. Twin births followed the randomisation process as per singleton births such that siblings would receive the same allocation.

### Imaging

Cranial ultrasound scans were performed by a single observer at 36 weeks’ CGA to measure the width of the subarachnoid space (primary outcome). Measurement of the subarachnoid space during routine cranial ultrasound was chosen as a pragmatic primary outcome measure performed by a single observer within a multicentre study to provide an indirect method of monitoring brain growth in preterm infants [24]. Occipito-frontal circumference at hospital discharge is associated with neurodevelopmental outcome [25]. We selected subarachnoid space as a surrogate outcome because it accounts for infants who have poor brain growth accompanied by high volumes of cerebrospinal fluid. Secondary outcomes were: (1) head circumference at 36 weeks’ CGA; (2) thyroid hormone ((thyroid stimulating hormone (TSH), FT4) plasma concentrations between birth and 36 weeks’ CGA; (3) auxological data (weight, lower leg length (knee to heel distance using knemometry), and occipito-frontal circumference); (4) survival; (5) duration of mechanical ventilation; and (6) chronic lung disease requiring home oxygen. All blood investigations and auxological measurements were made before supplementation (baseline) and on Day 14, Day 21, Day 28 and at 36 weeks’ CGA.

### Sample size calculation

Sample size was calculated using the standard deviation (SD) of subarachnoid space width using data from Armstrong *et al*. [24] Sixty-four infants in each group would have 80% power to detect a difference of 0.67 mm (that is, 0.5*SD) in subarachnoid space width between the groups at 36 weeks’ CGA. We assumed 20% loss to follow-up and aimed to randomize 150 infants. Analyses followed the principle of intention to treat with the allocation broken after determining inclusion in the analysis population for each outcome.

### Statistical analyses

A statistical analysis plan was agreed upon before the final analysis. Distributions of continuous outcomes were checked. *P* values were calculated using a *t*-test or Mann–Whitney *U* test as appropriate. Mixed models were used for longitudinal analyses, analysis of covariance to investigate leg length allowing for baseline measurements, and correlations used Spearman’s correlation. Analyses followed the principle of intention to treat with the allocation broken after determining inclusion in the analysis population for each outcome. Trial oversight was by an Independent Data and Safety Monitoring Committee (IDSMC). The IDSMC made recommendations to a Trial Steering Committee, which included a majority (three) of independent members. Quality control and assurance of the trial analyses and design of the trial were supported by the National Institute for Health Research Medicines for Children’s Clinical Trial Unit. The adoption of the study by the UK’s Medicines for Children’s Research Network supported coordination between centres [26] and the trial was subjected to inspection by the Medicines and Healthcare products Regulatory Association (MHRA).

### Ethical approval

The study was approved by North West Research Ethics Committee (reference number 07/MRE08/37) and by the Medicines for Human Regulatory Agency (MHRA). The parents of each potentially eligible baby were informed of the study’s objectives and overall requirements after birth when the baby had achieved respiratory and haemodynamic stability. The Investigator explained the study fully to the patient’s parent(s)/guardian(s) using a Patient Information Leaflet. The parent/guardian was then given at least 12 h to consider the study. If a parent/guardian was willing for the patient to participate in the study written informed consent was obtained.

## Results

**Figure 1:**
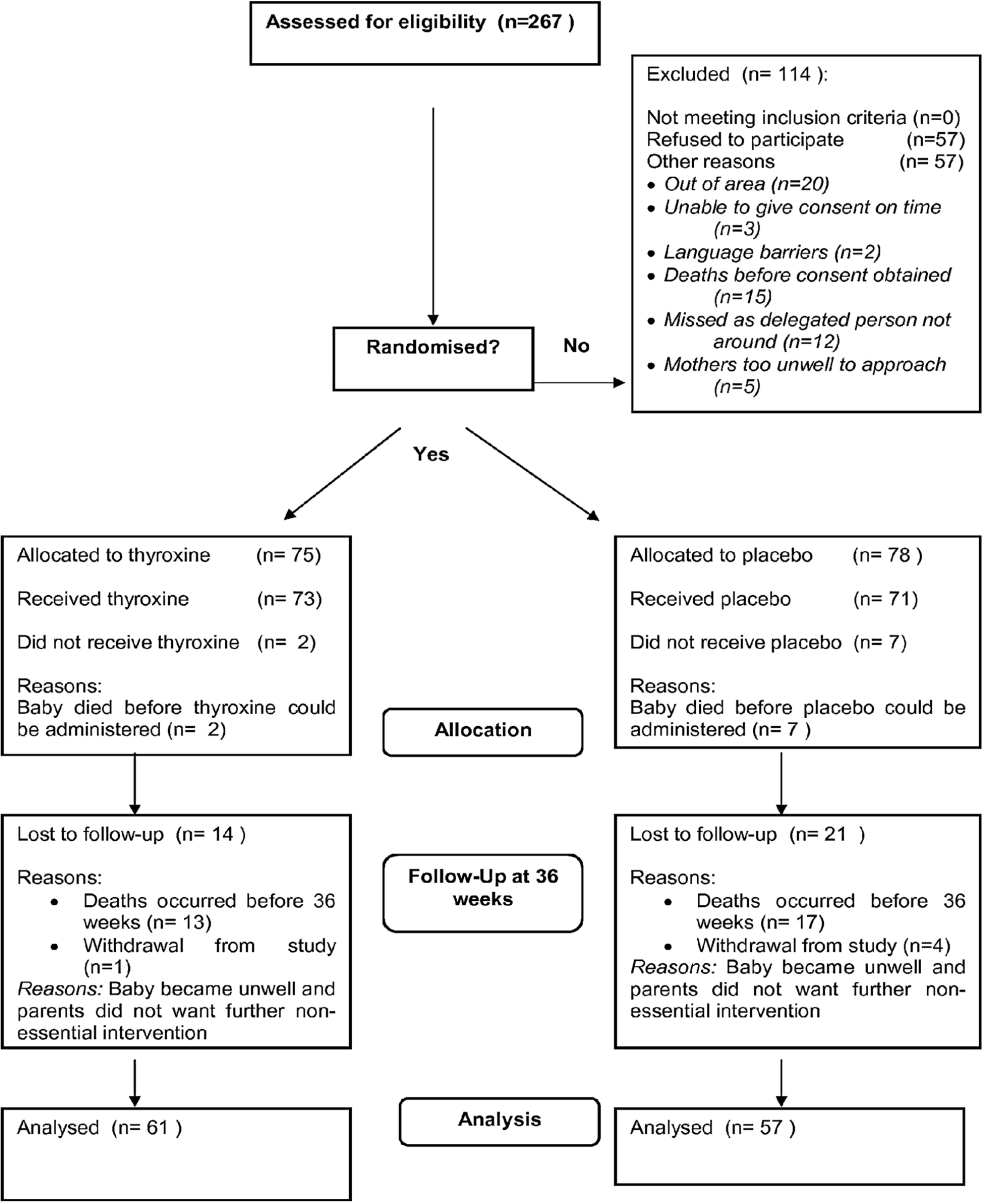
Consort diagram.

A total of 267 infants were assessed for eligibility from five recruiting centres and 153 infants were recruited to the trial. Recruited period commenced September 2007 and recruitment completed in June 2010. There were no differences in baseline characteristics between eligible infants recruited to the trial compared with infants not recruited to the trial. Seventy-eight infants received LT4 supplementation and 75 received placebo. Throughout the study no treatment allocation was unblinded and no suspected unexpected serious adverse reaction (SUSAR) was reported. The CONSORT flow diagram (Figure 1) shows the passage of all recruited and eligible participants.

Baseline characteristics between LT4-supplemented and placebo groups are shown in Table 1 and there were no significant differences between groups. The primary and secondary outcomes are shown in Table 2. Subarachnoid space width at 36 weeks’ CGA was similar for the two groups. Multivariate linear analysis of independent factors (gestational age, Clinical Risk Index for Babies (CRIB) score and mean FT4 levels) that might have affected the width of the subarachnoid space showed that mean FT4 levels were significant (beta=−0.37, *P*=0.03), that is, the lower the mean FT4 level, the wider the subarachnoid space. Gestation was the only independent factor affecting FT4 concentrations (beta 0.81, *P*=0.001).

**Table 1:** Demographics and baseline characteristics

	**LT4 -supplemented**	**Placebo**
	***n*= 75**	***n*= 78**
Gender (male)	44 (59%)	45 (58%)
Birth weight (grams)	820.7 (183.9)	842.2 (200.0)
Gestational age (weeks)	25.8 (1.3)	25.8 (1.4)
Clinical risk index for babies (CRIB) score	5.7 (3.5)	5.4 (4.1)
Twin births (pairs)	4 (5.3%)	6 (7.7%)
Infant baseline FT4 (pmol/L)	12.2 (6.9)	10.5 (6.3)
Infant baseline leg length (mm)	38.7 (7.1)	39.54 (6.8)
Maternal age (years)	28.8 (5.8)	28.9 (5.8)
Maternal history of smoking during pregnancy	16 (22%)	20 (26%)
Maternal history of alcohol during pregnancy	6 (8%)	4 (5%)
Maternal FT4 (pmol/L)	16.5 (3.2)	15.9 (2.5)
Premature rupture of membrane	30 (40.5%)	24 (30.8%)
Evidence of recent maternal infection^a^	21 (28.0%)	19 (24.4%)
Antenatal steroid therapy	72 (96.0%)	73 (93.6%)
Delivery by Caesarean section	24 (32.0%)	27 (35.1%)

Data expressed as mean (SD) for continuous outcomes, x (%) for categorical outcomes.

^a^Maternal infection was defined as maternal pyrexia >38°C or raised maternal blood C-reactive protein concentration during week before birth.

**Table 2:** Primary and secondary outcomes

	**LT4 -supplemented**	**Placebo**	**Difference (95% CI) LT4 - placebo**	***P *value**
Subarachnoid space width at 36 weeks’ CGA (cm)	0.20 (0.06)	0.21 (0.062	−0.01 (−0.03, 0.01)	0.34
	*n*=61	*n*=57		
Head circumference at 36 weeks’ CGA (cm)	30.40 (1.90)	30.61 (2.23)	−0.2 (−0.96, 0.54)	0.49
	*n*=61	*n*=57		
Leg length at 36 weeks’ CGA (mm)	61.52 (8.90)	65.20 (7.11)	−3.80 (−6.91, -0.62)	**0.02**
	*n*=53	*n*=53		
Mid arm circumference at 36 weeks’ CGA (mm)	8.03 (1.07)	8.19 (1.18)	−0.15 (−0.57, 0.25)	0.47
	*n*=60	*n*=57		
Weight at 36 weeks’ CGA (kg)	1.90 (0.40)	2.03 (0.40)	−0.11 (−0.26, 0.04)	0.14
	*n*=61	*n*=57		
Thyroid volume (mL) at 36 weeks’ CGA	0.57 (0.17)	0.57 (0.17)	0.004 (−0.07, 0.06)	0.99
	*n*=59	*n*=55		
Mortality	14 (18%)	19 (24%)	−0.06(−0.19, 0.08)	0.34
	*n*=75	*n*=78		
Duration of mechanical ventilation (days)	18.30 (13,38)	21.00 (8,42)	−2.00 (−5.00, 8.00)	0.56^a^
	*n*=58	*n*=58		
Days on total parenteral nutrition (TPN)	14.00 (10,21)	14.50 (11,21)	−1.00 (−4.00, 2.00)	0.59^a^
	*n*=58	*n*=58		
Chronic lung disease (CLD) diagnosed	42 (56%)	39 (50%)	0.06 (−0.10, 0.22)	0.46
	*n*=61	*n*=57		
Oxygen levels at 36 weeks’ CGA(L/min) for babies with CLD	0.50 (0.08, 1.5)	0.30 (0.08, 1.5)	0.00 (−0.17, 0.20)	0.99^a^
	*n*=61	*n*=57		

Data expressed as mean and standard deviation (SD) for continuous parametric outcomes and median and interquartile range (IQR) for non-parametric outcomes.

Student *t*-test for analysis of continuous parametric outcomes.

^a^Mann-Whitney Wilcoxon test for non-parametric outcomes.

The only secondary outcome difference was that infants who received LT4 had significantly shorter lower leg lengths at 36 weeks CGA than those in the placebo group. However, although the baseline mean lower leg length was not statistically significantly different, it trended longer in the placebo group than in the LT4 supplemented group (mean difference −0.83 mm (95%CI −3.35, 1.70, *P*=0.51)). Leg growth between baseline and 36 weeks CGA was similar for the two groups (mean difference −2.51 mm (95%CI −0.60 to 5.63), *P*=0.11), and adjustment for baseline values showed a mean difference that was not significant (−2.80 mm (95%CI −5.80, 0.19), *P*=0.06).

**Figure 2:**
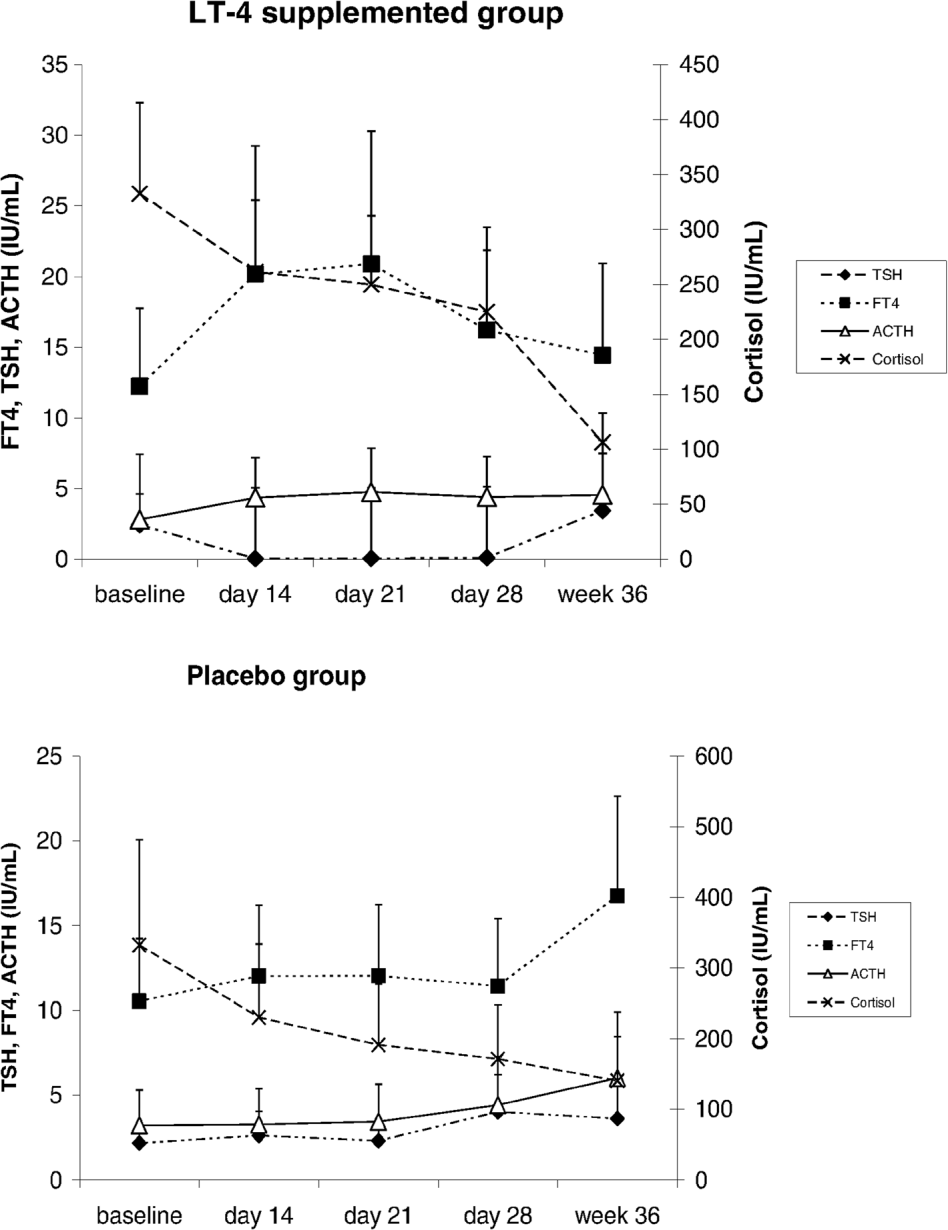
**Interactions between blood FT4, TSH, ACTH and cortisol concentrations.** The data points represent the mean value for each variable. ACTH, mean plasma ACTH concentration; Cort, mean plasma cortisol concentration; FT4, mean plasma FT4 concentration; TSH, mean plasma TSH concentration.

The plasma FT4 and TSH concentrations are shown in Table 3. The plasma FT4 hormone concentrations at baseline were similar for the two groups. Supplementation effectively significantly raised plasma FT4 concentrations in the LT4-supplemented group initially, but after stopping supplementation, plasma FT4 concentrations in the LT4-supplemented group became significantly lower than in the placebo group. Plasma cortisol levels for the two groups were similar. Figure 2 shows the longitudinal profiles of plasma FT4, TSH, cortisol and ACTH concentrations from baseline to week 36.

**Table 3:** Plasma thyroxine and TSH concentrations in the LT4-supplemented and placebo groups

	**TSH (mU/L)**			**FT4 (pmol/L)**		
	**LT4-supplemented**	**Placebo**	***P *value**	**LT4-supplemented**	**Placebo**	***P *value**
Baseline	2.44 (1.06,4.03)	2.15 (1.27,3.40)	0.62^a^	12.24 (6.93)	10.53 (6.34)	0.18
	*n*=43	*n*=53		*n*=53	*n*=54	
Day 14	0.05 (0.01,0.14)	2.60 (1.60,4.00)	**<0.001**^a^	20.17 (8.71)	12.01 (5.72)	**<0.001**
	*n*=42	*n*=37		*n*=44	*n*=38	
Day 21	0.06 (0.02,0.35)	2.29 (1.28, 2.76)	**<0.001**^a^	20.89 (9.43)	12.02 (5.73)	**<0.001**
	*n*=42	*n*=42		*n*=42	*n*=40	
Day 28	0.12 (0.01,1.44)	4.0 (2.22. 6.20)	**<0.001**^a^	16.18 (6.23)	11.42 (4.88)	**0.005**
	*n*=38	*n*=35		*n*=38	*n*=36	
Week 36	3.43 (1.82,4.90)	3.60 (2.10,4.70)	0.99^a^	14.44 (4.56)	16.76 (3.34)	**0.009**
	*n*=39	*n*=39		*n*=44	*n*=43	

Data expressed as mean and standard deviation (SD) for continuous parametric outcomes and median and interquartile ranges (IQR) for non-parametric outcomes.

Student *t*-test for analysis of continuous parametric outcomes.

^a^Mann-Whitney Wilcoxon test for non-parametric outcomes.

There were no significant adverse events noted in the LT4-supplemented or placebo groups.

## Discussion

The standard design of a randomised controlled trial is intended to evaluate the effectiveness of the introduction of a new technology to clinical practice, while explanatory trials allow for a better understanding of the mechanisms for any treatment effect found. This trial was designed with a surrogate outcome in order to indicate whether a trial of effectiveness is appropriate and as an explanatory trial [27] in order to improve understanding of the effects and mechanisms of thyroxine supplementation.

A daily supplement of LT4 until 32 weeks’ CGA raised plasma FT4 concentrations during the first 3 weeks after birth. TSH secretion in the LT4-supplemented group was suppressed by day 14 during supplementation, indicating some integrity of the HPT axis in these very immature babies. No documented adverse effects relating to signs or symptoms of hyperthyroidism were documented during the trial. After discontinuation of supplemental LT4, FT4 concentrations were significantly lower in babies who had received supplementation than those given placebo, suggesting an ability to respond to withdrawal of the external supply of hormone at least by 32 weeks’ CGA. Babies in the placebo group had increasing plasma FT4 concentrations between day 28 and week 36, in accordance with the natural history of this condition described previously [28].

Despite evidence that the dose used had an effect on the HPT axis, there was no difference between the LT4-supplemented infants and those in the placebo group with regard to the primary outcome of subarachnoid space width at 36 weeks’ gestation. There were no differences between the two groups for head circumference and weight at 36 weeks’ CGA.

Infants who received LT4 supplementation had significantly shorter lower leg lengths at 36 weeks’ CGA (*P*=0.02). However, after adjusting for baseline leg length, the difference failed to reach significance (*P*=0.06). This finding was unexpected. If it represents a real effect of LT4 supplementation, it is possible that higher FT4 levels early in life for LT4-supplemented babies caused an increase in metabolic requirements resulting in less good linear growth [9]. A possible mechanism is through the regulation of endochondral ossification, affecting skeletal development, linear growth and bone formation [29,30]. TSH, which appeared in this study to be downregulated by LT4-supplements, may also be a direct negative regulator of bone turnover, acting via the TSH receptor on both osteoblasts and osteoclasts [31].

There were no unexpected serious adverse events throughout the trial and no difference in the incidence of adverse events in the two groups. Our results concurred with van Wassenaer *et al.*[14] who also reported no significant difference in incidence of adverse events in their study.

A surrogate outcome (width of the subarachnoid space) was used to reflect brain size at 36 weeks’ CGA although there are no data relating ultrasound measured width of subarachnoid space with neurodevelopmental outcome. However, head size, and therefore brain size, at term-equivalence is associated with long-term neurodevelopmentalal outcome in extremely preterm neonates so that this surrogate outcome has some validity and has been partially qualified as a surrogate outcome [24,32]. It is also possible that this outcome failed to detect an important difference between the groups. Brain MRI may provide a more relevant surrogate. However, experience with a previous trial involving a similar group of babies and parents indicated that consent to MRI scans in this population is relatively low [33]. Our experience confirmed this: the parents of only 38% of surviving infants consented to the TIPIT MRI sub-study. Participants in this trial are currently receiving neurodevelopmental follow-up at 42 months of age.

LT4-supplementation increases the metabolism of cortisol to biologically inactive cortisone and might lead to significant adrenal insufficiency [34]. However, there was no evidence of worsening plasma cortisol levels with LT4 supplementation. Our findings were concordant with a study by Valerio *et al.* who showed that T(3) and T(4) administration did not have any effect on cortisol levels [19].

It remains unclear whether low levels of circulating thyroxine are physiologically appropriate for such very immature babies, or whether THOP is a kind of transient hypothyroidism that is centrally mediated. Therefore, the effects of ‘correction’ of the postnatal FT4 concentrations in the individual premature infant are difficult to predict. It is impossible to predict the severity and duration of the low FT4 state in an individual. It is also not possible to propose a ‘one dose fits all’ approach to normalise the FT4 as it is difficult to estimate the optimal FT4 concentrations for an extremely premature infant without measuring serial FT4 and TSH concentrations. Our results do show significant correlations between low FT4 concentrations and wider subarachnoid space, that is, smaller brain size. It is therefore plausible that it is the actual FT4 concentrations achieved that creates an impact on central nervous system development.

## Conclusions

The findings from this study suggest that giving supplementary thyroxine to all babies below 28 weeks’ gestation does not improve short-term outcome. It appears that gestational age alone is not a sufficiently strong risk factor to institute thyroid supplementation for all preterm infants or to use as an inclusion criterion in RCTs. This study suggests that further studies of this intervention should target infants with low FT4 levels, and other approaches to THOP may also be usefully investigated.

## Competing interests

The authors declare that they have no competing interests.

## Authors’ contributions

SMN, MAT and AMW conceived the study, participated in its design and coordination and drafted the manuscript. MD, SV, RG, DM, PS and PN participated in the design of the study. CG participated in the design of the study and the statistical analysis. All authors read and approved the final manuscript.

## References

[B1] Salerno M Militerni R Di Maio S Bravaccio C Gasparini N Tenore A Intellectual outcome at 12 years of age in congenital hypothyroidism Eur J Endocrinol 1999 141 105 110 10.1530/eje.0.1410105 10427151

[B2] Fisher DA Klein AH Thyroid development and disorders of thyroid function in the newborn N Engl J Med 1981 304 702 712 10.1056/NEJM198103193041205 6258072

[B3] Song SI Daneman D Rovet J The influence of etiology and treatment factors on intellectual outcome in congenital hypothyroidism J Dev Behav Pediatr 2001 22 376 384 10.1097/00004703-200112000-00005 11773802

[B4] Williams FL Visser TJ Hume R Transient hypothyroxinaemia in preterm infants Early Hum Dev 2006 82 797 802 10.1016/j.earlhumdev.2006.09.007 17079099

[B5] Biswas S Buffery J Enoch H Bland JM Walters D Markiewicz M A longitudinal assessment of thyroid hormone concentrations in preterm infants younger than 30 weeks’ gestation during the first 2 weeks of life and their relationship to outcome Pediatrics 2002 109 222 227 10.1542/peds.109.2.222 11826199

[B6] Chowdhry P Scanlon JW Auerbach R Abbassi V Results of controlled double-blind study of thyroid replacement in very low-birth-weight premature infants with hypothyroxinemia Pediatrics 1984 73 301 305 6366725

[B7] Williams FL Simpson J Delahunty C Ogston SA Bongers-Schokking JJ Murphy N van Toor H Wu SY Visser TJ Hume R Developmental trends in cord and postpartum serum thyroid hormones in preterm infants J Clin Endocrinol Metab 2004 89 5314 5320 10.1210/jc.2004-0869 15531476

[B8] van Wassenaer AG Kok JH Dekker FW de Vijlder JJ Thyroid function in very preterm infants: influences of gestational age and disease Pediatr Res 1997 42 604 609 10.1203/00006450-199711000-00009 9357931

[B9] LaFranchi S Thyroid function in the preterm infant Thyroid 1999 9 71 78 10.1089/thy.1999.9.71 10037080

[B10] Lucas A Morley R Fewtrell MS Low triiodothyronine concentration in preterm infants and subsequent intelligence quotient (IQ) at 8 year follow up BMJ 1996 312 1132 1133 discussion 1133–1134 10.1136/bmj.312.7039.1132 8620130 PMC2350658

[B11] Lucas A Rennie J Baker BA Morley R Low plasma triiodothyronine concentrations and outcome in preterm infants Arch Dis Child 1988 63 1201 1206 10.1136/adc.63.10.1201 2461683 PMC1779041

[B12] Meijer WJ Verloove-Vanhorick SP Brand R van den Brande JL Transient hypothyroxinaemia associated with developmental delay in very preterm infants Arch Dis Child 1992 67 944 947 10.1136/adc.67.7.944 1381573 PMC1793825

[B13] Vanhole C Aerssens P Naulaers G Casneuf A Devlieger H Van den Berghe G de Zegher F L-thyroxine treatment of preterm newborns: clinical and endocrine effects Pediatr Res 1997 42 87 92 10.1203/00006450-199707000-00014 9212042

[B14] van Wassenaer AG Kok JH de Vijlder JJ Briet JM Smit BJ Tamminga P van Baar A Dekker FW Vulsma T Effects of thyroxine supplementation on neurologic development in infants born at less than 30 weeks’ gestation N Engl J Med 1997 336 21 26 10.1056/NEJM199701023360104 8970936

[B15] Paneth N Does transient hypothyroxinemia cause abnormal neurodevelopment in premature infants? Clin Perinatol 1998 25 627 643 9779338

[B16] Osborn D Hunt R Prophylactic postnatal thyroid hormones for prevention of morbidity and mortality in preterm infants Cochrane Database Syst Rev 2007 1 CD005948 17253571 10.1002/14651858.CD005948.pub2 PMC9004229

[B17] Briët JM Van Wassenaer AG Dekker FW De Vijlder JJM Van Baar A Kok JH Neonatal thyroxine supplementation in very preterm children: developmental outcome evaluated at early school age Pediatrics 2001 107 712 718 10.1542/peds.107.4.712 11335749

[B18] Smith LM Leake RD Berman N Villanueva S Brasel JA Postnatal thyroxine supplementation in infants less than 32 weeks’ gestation: effects on pulmonary morbidity J Perinatol 2000 20 427 431 10.1038/sj.jp.7200417 11076326

[B19] Valerio PG van Wassenaer AG de Vijlder JJ Kok JH A randomized, masked study of triiodothyronine plus thyroxine administration in preterm infants less than 28 weeks of gestational age: hormonal and clinical effects Pediatr Res 2004 55 248 253 10.1203/01.PDR.0000104153.72572.F5 14630985

[B20] Sackett DL Explanatory and pragmatic clinical trials: a primer and application to a recent asthma trial Pol Arch Med Wewn 2011 121 259 262 21878863

[B21] Micheal W Clinical aspects of brain injury in the preterm infant The Newborn Brain: neuroscience and clinical applications 2010 2 Cambridge: Cambridge University Press

[B22] Ng SM Turner MA Gamble C Didi M Victor S Weindling AM TIPIT: a randomised controlled trial of thyroxine in preterm infants under 28 weeks’ gestation Trials 2008 9 17 10.1186/1745-6215-9-17 18366798 PMC2335090

[B23] van Wassenaer AG Kok JH Endert E Vulsma T de Vijlder JJ Thyroxine administration to infants of less than 30 weeks’ gestational age does not increase plasma triiodothyronine concentrations Acta Endocrinol (Copenh) 1993 129 139 146 8372599 10.1530/acta.0.1290139

[B24] Armstrong DL Bagnall C Harding JE Teele RL Measurement of the subarachnoid space by ultrasound in preterm infants Arch Dis Child Fetal Neonatal Ed 2002 86 F124 F126 10.1136/fn.86.2.F124 11882556 PMC1721376

[B25] Cooke RW Perinatal and postnatal factors in very preterm infants and subsequent cognitive and motor abilities Arch Dis Child Fetal Neonatal Ed 2005 90 F60 F63 10.1136/adc.2004.059188 15613579 PMC1721829

[B26] Ng SM Weindling AM The impact of networks on clinical trials in the United Kingdom Trials 2009 10 100 10.1186/1745-6215-10-100 19889222 PMC2775734

[B27] McMahon AD Study control, violators, inclusion criteria and defining explanatory and pragmatic trials Stat Med 2002 21 1365 1376 10.1002/sim.1120 12185890

[B28] van Wassenaer AG Kok JH Dekker FW Endert E de Vijlder JJ Thyroxine administration to infants of less than 30 weeks gestational age decreases plasma tri-iodothyronine concentrations Eur J Endocrinol 1998 139 508 515 10.1530/eje.0.1390508 9849815

[B29] Shao YY Wang L Ballock RT Thyroid hormone and the growth plate Rev Endocr Metab Disord 2006 7 265 271 17200892 10.1007/s11154-006-9012-2

[B30] Kisakol G Kaya A Gonen S Tunc R Bone and calcium metabolism in subclinical autoimmune hyperthyroidism and hypothyroidism Endocr J 2003 50 657 661 10.1507/endocrj.50.657 14709834

[B31] Galliford TM Murphy E Williams AJ Bassett JH Williams GR Effects of thyroid status on bone metabolism: a primary role for thyroid stimulating hormone or thyroid hormone? Minerva Endocrinol 2005 30 237 246 16319811

[B32] Cooke RW Are there critical periods for brain growth in children born preterm? Arch Dis Child Fetal Neonatal Ed 2006 91 F17 F20 16223756 10.1136/adc.2005.077438 PMC2672640

[B33] Tan M Abernethy L Cooke R Improving head growth in preterm infants–a randomised controlled trial II: MRI and developmental outcomes in the first year Arch Dis Child Fetal Neonatal Ed 2008 93 F342 F346 10.1136/adc.2007.124255 18285378

[B34] Scott SM Cimino DF Evidence for developmental hypopituitarism in ill preterm infants J Perinatol 2004 24 429 434 10.1038/sj.jp.7211112 15129226

